# Effects of Dog-Assisted Therapy in Adolescents with Eating Disorders: A Study Protocol for a Pilot Controlled Trial

**DOI:** 10.3390/ani11102784

**Published:** 2021-09-24

**Authors:** Ana Myriam Lavín-Pérez, Cristina Martín-Sánchez, Beatriz Martínez-Núñez, Luis Lucio Lobato-Rincón, Santos Villafaina, Israel González-García, Ana Mata-Cantero, Montserrat Graell, Eugenio Merellano-Navarro, Daniel Collado-Mateo

**Affiliations:** 1 Centre for Sport Studies, Rey Juan Carlos University, Fuenlabrada, 28943 Madrid, Spain; am.lavin.2018@alumnos.urjc.es (A.M.L.-P.); daniel.collado@urjc.es (D.C.-M.); 2 Animal-Assisted Intervention Office, Rey Juan Carlos University, Móstoles, 28933 Madrid, Spain; c.martinsanch@alumnos.urjc.es (C.M.-S.); luislucio.lobato@urjc.es (L.L.L.-R.); oficina.iaa@urjc.es (I.G.-G.); 3 Psychiatry and Clinical Psychology Department, Hospital Infantil Universitario Niño Jesús, 28943 Madrid, Spain; beamartinezn@gmail.com (B.M.-N.); montserratgraell1@gmail.com (M.G.); 4 Department of Psychology, King Juan Carlos University, Alcorcón, 28943 Madrid, Spain; 5 Physical Activity and Quality of Life Research Group (AFYCAV), Faculty of Sport Sciences, University of Extremadura, 10003 Caceres, Spain; 6 Perroterapia-Animal-Assisted Interventions, Rivas Vaciamadrid, 28943 Madrid, Spain; ana.mata@perroterapia.org; 7 Centro de Investigación Biomédica en Red de Salud Mental (CIBERSAM), 28943 Madrid, Spain; 8 Grupo de Investigacion EFISAL, Universidad Autónoma de Chile, 3460000 Talca, Chile

**Keywords:** animal-assisted therapies, anorexia nervosa, bulimia nervosa, mental health

## Abstract

**Simple Summary:**

Animal-assisted therapies may lead to benefits in anxiety, depression, low self-esteem, or quality of life. These variables are commonly present among patients with eating disorders. Thus, the current pilot study will aim to evaluate the effects of a dog-assisted therapy on the eating disorders symptoms, mental, psychosocial, and physical health, quality of life, and handgrip strength of adolescents suffering from eating disorders. Thirty-two patients will participate and 16 of them will receive the intervention while the other 16 will be the control group. Both groups will continue with their treatments prescribed at the hospital, but the experimental group will participate in a dog-assisted therapy program involving 7 sessions in 7 weeks. This will be the first study to examine the effects of dog-assisted therapy in this population. Significant improvements, in the primary and secondary outcomes, may be expected based on the known benefits of AAT on self-esteem, stress, and self-control in different populations. Finally, although the program is focused on the improvement of adolescents’ health, animal welfare will be a priority in this study.

**Abstract:**

Background: Eating disorders are characterized by a persistent disturbance that alters food intake and it is often accompanied by anxiety, depression, low self-esteem, or reduced functional capacity and quality of life. Animal-assisted therapies (AAT) have shown benefits in these variables in children and adult populations. Thus, the present pilot study will aim to evaluate the effects of a dog-assisted therapy on the eating disorders symptoms, mental, psychosocial, and physical health, quality of life, and handgrip strength of adolescents suffering from eating disorders. Methods: The current pilot study will involve 32 patients, who will be assigned to a control or an experimental group. Intervention will be conducted once a week for seven weeks. Neither the experimental nor the control group will discontinue their usual care. The main outcome measures will be the eating disorder symptoms and the health-related quality of life measured with standardized questionnaires, while the secondary variables will be anxiety, depression, character, behavior, strength, and body mass. Conclusions: This pilot-controlled trial will be the first to evaluate the effects of dog-assisted therapy on the physical and mental health of adolescents with eating disorders. Significant improvements, in the primary and secondary outcomes, may be expected based on the known benefits of AAT on self-esteem, stress, and self-control in different populations. Finally, although the program is focused on the improvement of adolescents’ health, animal welfare will be a priority in this study.

## 1. Introduction

The DSM-5 [1] classifies the different eating and feeding disorders. They are characterized by a persistent disturbance in eating or eating-directed behavior, which leads to altered food consumption or absorption and causes a deterioration of physical health and psychosocial functioning [1]. The etiology of eating disorders is multifactorial, including both biological (genetic causes or abnormal neurotransmitter systems) and psychological risk factors such as low self-esteem, body dissatisfaction or obsessive thinking [2,3,4]. Most common eating disorders are anorexia nervosa and bulimia nervosa [1], ranged from 1% to 4% of European women [5]. Worldwide, anorexia nervosa and bulimia nervosa had a prevalence of 176.2 per 100,000 people [6]. Among adolescents, the prevalence ranged from 0.3% to 2.2% for anorexia nervosa and 0.1% to 2% for bulimia nervosa [7]. However, epidemiological studies have been focused on bulimia nervosa and anorexia nervosa, omitting other eating disorders such as binge-eating disorder and other specified feeding or eating disorders [6,8,9]. This is relevant, as it is estimated that 17.3 million people suffered from binge-eating disorder and 24.6 million people suffered from other specified feeding or eating disorder [10].

The treatment for eating disorders is challenging due to the large individual patients’ differences and thoughts [11]. Thus, it is recommended a multidisciplinary treatment, including family-based, psychological, and pharmacological approaches [4]. Some of the symptoms associated with eating disorders have been previously treated through Animal Assisted Therapy (AAT) in studies conducted with different populations and diseases, such as attention-deficit/hyperactivity disorder, anxiety or depression, to enhance psychological and social factors [12,13,14,15]. The International Association of Human-Animal Organizations (IAHAIO) defines AAT as a goal-oriented, planned and structured therapeutic intervention directed and/or delivered by health, education or human service professionals [16].

Previous studies [15,17] showed a significant relationship between AAT sessions and self-esteem improvements and reduced anxiety levels [18,19,20]. Regarding social skills, Whitely [21] and Duckers [22] have shown that AAT sessions, together with teamwork, could improve social skills and contribute to the development of problem-solving strategies. In the case of interventions focused on improving depression (Francis et al., 1985; Souter and Miller, 2007; Veilleux, 2020), AAT showed a significant reduction of depressive symptoms as patient’s rediscovered joy, spontaneity, and healthy leisure capacity through the animal, reducing anhedonia. Moreover, improvements in cooperation and self-control and reduction of the patient’s anger, agitation, and aggressiveness may be achieved due to the need to communicate with the animal calmly and non-reactive, promoting emotional skills, impulse regulation, and self-control [13,23,24]. A previous systematic review hypothesized that the benefits obtained by AAT in psychological health could be due to the liberation of oxytocin [25]. Nevertheless, the results are contradictory [26]. Future studies are encouraged to examine the mechanisms under the effects of AAT on psychological and physiological domains.

Due to the benefits mentioned above and considering that AAT tried to minimize infection risk [27,28] following the current legislation [29], AAT could be a potentially effective tool to improve adolescents’ physical and mental health with eating disorders. This would be relevant, as adolescence is a critical age in the development of self-esteem and self-concept [30,31], in which low values of these variables may be related to the development of eating disorders [32,33]. Thus, the present pilot study will propose a dog-assisted therapy program in a population of adolescents with eating disorders to improve the main associated symptoms and consequences of the disorder. The current pilot design has been chosen to explore the feasibility of an AAT intervention in adolescents with eating disorders. Nevertheless, considering the absence of scientific literature in the field and the study design of the current pilot study, results would be relevant in the field of eating disorders and AAT.

### Objectives and Hypothesis

The main objective of the current pilot parallel controlled clinical trial is to explore the feasibility of an AAT intervention to improve the eating disorder symptoms, the health-related quality of life anxiety, depression, adolescent character and behavior, strength, body mass and treatment satisfaction of adolescents with eating disorders through dog-assisted therapy in contrast to a control group. Secondarily, this study also aimed to provide the results on the effects on anxiety, depression, adolescent character and behavior, strength, body mass and treatment satisfaction. However, it must be noted that, as a pilot study, results must be interpreted with caution.

We hypothesized that adolescents who participate in the AAT (dog-assisted therapy) would improve eating disorder symptoms, quality of life and mood while reducing the anxiety and depressive symptoms and their behavioral problems compared to the control group. Handgrip strength could also be improved as a physical manifestation of overall health status [34]. Furthermore, this test has been conducted in eating disorders due to the importance of strength to avoid sarcopenia [35] as well as to evaluate the physical risk [36]. Besides, the treatment satisfaction is expected to be favorable to patients, families, and the medical staff from the hospital.

## 2. Materials and Methods

### 2.1. Study Design

The current pilot study, a parallel-group non-randomized clinical trial, aimed to analyze the effectiveness of a dog-assisted therapy in adolescents with eating disorders compared to the control group that will receive their usual care. The study setting where the intervention will be carried out is the Niño Jesús University Children’s Hospital (Psychiatry and Clinical Psychology Department) in Madrid, Spain. This study protocol followed the SPIRIT 2013 Statement Items to correctly fulfil the standard protocol items for clinical trials [37]. If during the study process any substantial modification is needed, the relevant parties involved will be informed (investigators, hospital’s ethics committee, and trial registration platform).

### 2.2. Ethical Approval and Trial Registration

The protocol has been approved by the Ethics Committee of the Niño Jesús University Children’s Hospital in March 2021 with the approval number of R-0007/21. After the positive response, the trial was registered at ClinicalTrials.gov under the identification number NCT04869423.

### 2.3. Participants

#### 2.3.1. Eligibility Criteria

Participants will need to fulfil the following inclusion criteria in order to be included in the study: (a) adolescents aged <18 (b) treated in the Day Hospital of the Psychiatry and Psychology Service of the Niño Jesús Children’s University Hospital, (c) to have been diagnosed with any kind of eating disorder (anorexia nervosa, bulimia nervosa, binge eating or other specified feeding or eating disorder) according to the DSM-5 criteria, (d) adolescents willing to participate and available to assist, (e) having read, agreed and signed the written informed consent by patients and their legal guardians. Moreover, patients with dog allergy, dog phobia and history of impulsive animal aggression will be excluded from the study.

#### 2.3.2. Recruitment

Initial recruitment, screening, and baseline assessment for the study will be conducted between April and October 2021. The participants’ enrollment will be voluntary, and the day hospital patients from the Niño Jesús University Children’s Hospital will offer them to be part of the study. If they are interested, the detailed information of the study will be explained to their legal guardians so they can approve, by signing the informed consent, the adolescents’ participation. Participation in this study is voluntary, and the involvement in the AAT group is conditioned by medical criteria, time period, and the availability to attend the sessions. Thus, the recruitment procedure is resulting in a sample of opportunity.

#### 2.3.3. Randomization and Blinding

The current pilot study is a non-randomized pilot-controlled trial. Although participants will know their group assignment, the evaluators will be blinded to the assignment. An anonymous identification code will be assigned to each participant to preserve confidentiality so that the evaluators will not know patients’ personal data or recognize the allocation group.

### 2.4. Intervention

The intervention will last nine weeks, including one evaluation session in the first week, seven intervention (or usual care) weeks, and another evaluation session in the last week. Given that there are no previous studies with similar aims, this duration was set as a consensus among all the researchers involved in the study, including the medical staff from the psychiatry unit, the professional handlers, and the academics.

Dogs will follow a strict zoonosis protocol, including behavioral, blood, urine, and feces analyses. Deworming, rabies, and tetravalent vaccinations were also required. Animal welfare will be ensured by the Animal Assisted Intervention Office of the University through a protocol that includes not only the physical health of the animal but also tries to reduce or avoid the presence of fatigue or stress during AAT sessions and also during training. Thus, the handler and the dog must have a strong mutual bond so the handler can rapidly and adequately interpret the dog’s body language and identify potential stressful or uncomfortable situations. Any session can be interrupted, and any activity can be skipped if the handler thinks that his/her dog is not having a good time.

#### 2.4.1. Experimental Group

The planned intervention will have a length of seven weeks, where patients will participate in a dog-assisted therapy once per week (seven sessions in total). The intervention will be added to the usual care they will be receiving at the Hospital, so they will not have to discontinue any treatment. In order to have complete control of the adolescents and the dogs, the therapy will be carried out in small groups composed of four patients.

Adolescents who will be discharged from the Hospital will have the opportunity to continue with the intervention. If during the intervention any patient presents an allergy or had aggressive behavior toward the dog, the expert will exclude the participant from the experimental group. Besides participating in the dog-assisted therapy program, patients will continue with their usual hospital and/or pharmacological treatments.

As AAT should be directed by health professionals, the sessions will be directed by a psychologist accompanied by two dog handlers. Dog handlers’ responsibilities are: (a) to be responsible at all times for the dog’s interaction; (b) to identify him/herself and the dog before accessing at the hospital; (c) to ensure dog hygiene and well-being conditions; and (d) to provide necessary material for the development of the activity.

**Figure 1:**
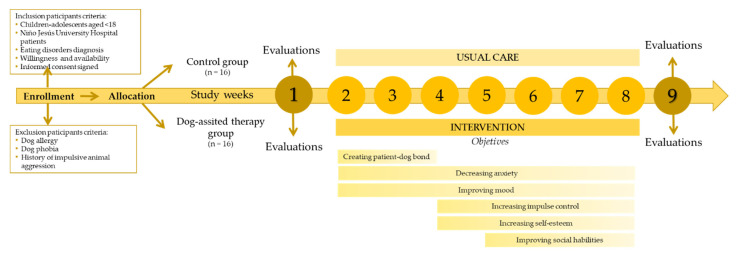
Study timeline and intervention objectives distribution during the dog-assisted therapy sessions.

The planned sessions, with a duration of 50 min, will include a welcome part aimed to get in touch with the dog (5 min), a main part where participants will learn basic notions about dog training and then try to train the dogs (40 min) and a closing part to say goodbye to the dogs (5 min). In the main part, patients will perform different activities and exercises with the dog to progressively work the following objectives to improve patients mental and physical health. The intervention will include the following objectives (two or more different aims can be addressed in the same session): (1) creating a patient-dog bond (2 sessions); (2) decreasing anxiety (7 sessions); (3) improving mood (7 sessions); (4) improve social abilities (4 sessions); (5) increasing impulse control (5 sessions); and, (6) enhance self-esteem (5 sessions) (See Figure 1).

In this regard, in the first two weeks of the interventions, we could find activities where adolescents will interact with dogs freely, as well as activities where they will have to groom the dogs. Furthermore, in these sessions, adolescents would learn different training techniques and skills that they can use with the dogs, as well as they, will know the importance of therapy dogs in different institutions and patients. These activities will create a patient-dog bond. After this introductory period, in the following five weeks, participants will perform activities of dog training where adolescents, for instance, must choose a behavior to teach the dog between different options, show the training results to the other participants or even play a question-and-answer game where the success depends on the bond between the dogs and the adolescents. The program was designed to maximize the physical contact with the animal (always according and considering the preferences of the participants), as it may be one of the main aspects contributing to the effectiveness of AAI programs [38].

#### 2.4.2. Control Group

The control group will have the same inclusion and exclusion criteria as the experimental group. This group will continue with the treatment carried out within the Spanish Public Health System (usual care) provided by the Niño Jesús University Children’s Hospital. This usual care may involve pharmacological and non-pharmacological treatments.

### 2.5. Measures and Procedures of the Outcome Measures

All the assessments will be carried out in the Psychiatry and Clinical Psychology Department of the Niño Jesús University Children’s Hospital. Participants’ sociodemographic information will be collected. Variables such as age, gender, patients’ and parents’ country, place of residence, or educational level will be asked. Moreover, information regarding disease diagnosis (type of eating disorder and time from diagnosis), pharmacological treatment, and current and/or former animals at home will be collected.

#### 2.5.1. Eating Disorder Symptoms

The Spanish version of the Eating Disorder Inventory (EDI-2) [39] will be employed to evaluate the evolution of anorexia nervosa and bulimia nervosa symptoms [40]. The EDI-2 is a 91-item scale with 11 subscales (Drive for Thinness, Bulimia, Body Dissatisfaction, Ineffectiveness, Perfectionism, Interpersonal Distrust, Interoceptive Awareness, Maturity Fears, Asceticism, Impulse Regulation, Social Insecurity). Responses are represented in a Likert scale from 5 (usually) to 0 (never). Higher scores mean more severe symptoms. Thus, total score represents the outcome measure for eating disorder symptoms.

#### 2.5.2. Health-Related Quality of Life

Health-related quality of life will be assessed with the Kidscreen-10 Index [41]. It will allow comparing patients’ results to the common population average which provides the limit to classify the results as “normal” (when the values are in the average) or “sensible” (if the scores are below the average). The measure tool consists of 10 items. This index has a high internal consistency (Cronbach’s α of 0.82) and good retest reliability (*r* = 0.73; ICC = 0.72). Scores can range between 10 and 50, with higher score meaning better health-related quality of life [41].

#### 2.5.3. Anxiety

The State-Trait Anxiety Inventory for children (STAI-C) questionnaire will be used to assess the adolescents’ anxiety [42]. The Spanish version, composed of two different scales, one to assess state anxiety (20 items) and the other to evaluate trait anxiety (20 items) in adolescents [43] will be employed. Each item is rated from 1 to 3, so the sub-scale scores can range from 20 to 60. Higher scores are interpreted as higher anxiety [43].

#### 2.5.4. Depression

Depression will be measured by the Children Depression Inventory (CDI) [44]. This questionnaire includes 27 items with three possible options from 0 (absence of symptoms) to 2 (severe symptoms). For the current study, the validated Spanish version will be used [45]. The total score ranges from 0–54, with higher scores being interpreted as higher depression. This scale had a Cronbach’s α between 0.75 and 0.94 and a test reliability of 0.84.

#### 2.5.5. Adolescent Character

The Spanish version of the Temperament and Character Inventory—Revised [46] will be employed to assess the patients’ character [47]. It consists of 240 items and 5 options for each one. It measures 4 temperaments, Novelty Seeking (NS), Harm Avoidance (HA), Reward Dependence (RD), and Persistence (PS), and three characters, Self-directedness (SD), Cooperativeness (CO), and Self-transcendence (ST). Higher scores mean higher levels in each dimension.

#### 2.5.6. Adolescent Behavior

To evaluate the patients’ behavior, the Spanish Child Behavior Checklist inside the Achenbach System of Empirically Based Assessment will be used [42,48]. A total of 113 items conformed the scale. In order to get detailed information of participants behaviors, items are grouped in the following 8 subdimensions: anxiety-depression, isolation-depression, somatic complaints, social problems, thinking problems, attentional problems, break the rules conduct, and aggressive behavior. It is scored from 0 = “not true” to 2 = “true”. Scores range from 0 to 226. Higher scores mean a worse outcome [48].

#### 2.5.7. Strength

Patients’ strength will be assessed through the isometric maximum strength using a handgrip dynamometer (Takei TKK 5401 Digital Handgrip Dynamometer, Tokyo, Japan). Adolescents will have to press with the hand while it is entirely straight [35]. This test has been employed before in anorexia nervosa patients due to the importance of strength to avoid sarcopenia in this population [35].

#### 2.5.8. Body Mass Index

Adolescents’ weight and height will be assessed by using a SECA weighing device (SECA, Hamburg, Germany). Body mass index (BMI) will be calculated as follow: BMI = weight (kg) ÷ height^2^ (m).

#### 2.5.9. Treatment Satisfaction

Treatment satisfaction will be evaluated by the treatment satisfaction scale (CRES-4) Feixas i Viaplana, et al. [49]. This questionnaire has three dimensions: satisfaction (general satisfaction with the way the therapist dealt with the problem), problem-solution (how the treatment has helped in relation to the problem) and perception of emotional change (self-perception about the improvements in the emotional state after the treatment). The score in each dimension ranges from 0 to 100 and the global score is the sum of the dimensions (from 0 to 300). Higher scores mean higher satisfaction [49]. Furthermore, a short interview will be answered by participants once the intervention is finished in order to know their opinion (strengths and limitations) of the sessions and the potential benefits.

### 2.6. Data Analysis

All the outcome results will be included in an anonymous database to conduct the statistical analyses. A descriptive, quantitative, and graphics analysis will be performed of all the included variables. The statistic software employed will be the IBM Statistical Package for the Social Sciences (version 25.0; SPSS, Inc., Chicago, IL, USA) [50].

First of all, the Kolmogorov–Smirnov and Shapiro–Wilk tests will be performed to decide the implementation of parametric or non-parametric statistical analyses. Due to the nature of the study (a pilot study) and the low sample size, non-parametric statistical analyses emerged as the most adequate approach.

In this regard, as non-parametric statistics will be performed, the change from baseline for each variable will be calculated and the Mann–Whitney test performed and we will calculate the r as effect size [51]. Therefore, results will include medians, interquartile ranges, the effect size and the statistical significance (obtained from Mann–Whitney U test) for each primary and secondary outcome. Due to the large number of planned analyses and the low sample size, the alpha level of significance will be adjusted according to the Benjamini–Hochberg procedure to avoid type I error derived from multiple comparisons [52].

Additionally, if all variables were normally distributed, parametric statistics could be also performed and results could be reported as means and standard deviations. Furthermore, the therapy’s effects would be assessed using a repeated-measures MANOVA, and the partial eta squared effect size calculated.

## 3. Discussion

The present pilot study will be the first one evaluating the effects a parallel pilot-controlled trial based on dog-assisted therapy in adolescents with eating disorders. Two groups will compose the study: an AAT group and a control group. The AAT will last seven weeks, and one psychologist and two dog-handlers will direct it. Control group will continue with the usual care provided by the Psychiatry and Clinical Psychology Department of the Niño Jesús University Children’s Hospital. We will assess Eating disorder symptoms, Health-related quality of life, Anxiety, Depression, Adolescent character and behavior, Strength, Body mass and treatment satisfaction before and after the intervention.

The future study has been designed as a pilot study where we will assess the feasibility and usefulness of the AAT intervention in adolescents with eating disorders. This study design has been chosen because it is the first time that Niño Jesús University Children’s Hospital was involved in an animal-assisted therapy. Therefore, it is a great opportunity for health professional, adolescents and parents to explore the possible strengths and limitations of this type of interventions. Taking into account the strengths and limitations, a future randomized controlled trial will be performed, increasing the sample size and the duration of the therapy. Thus, as a pilot study, the results must be interpreted with caution, and future studies must confirm the findings of this trial.

As commented above, this will be the first study to evaluate the effects of an AAT using therapy dogs in children or adolescents suffering from eating disorders [53], examining physiological, behavioral, social, and physical variables. The measurement of these variables will provide a global perspective of the potential benefits of AAT on the health of adolescents with eating disorders. In this regard, previous studies have evaluated the effects of equine-assisted therapy [54] and dolphin therapies [55] in a population comprised of adult women but not in adolescents. Importantly, AAT seems to improve self-esteem in children and adolescents [15,56]. Although the exact mechanisms behind self-esteem improvement after AAT are unknown [15], this is relevant as self-esteem improvement is one of the challenges in eating-disorder treatment [57].

We hypothesized that adolescents who participated in the AAT group would enhance the eating disorder symptoms, quality of life and mood while reducing the anxiety and depressive symptoms and their behavioral problems compared to the control group. In this regard, previous studies have reported improvements in health-related quality of life, anxiety, depression or mood after AAT. This is relevant because all these symptoms are present in people with eating disorders [58,59,60]. Improvements could be due to calming or stress-relieving [61] that the presence of animals could induce, which is based on the biophilia hypothesis [62]. Furthermore, physical contact has been identified as one of the main mechanisms to achieve relevant benefits from AAT, such as stress reduction or the feeling of being connected and not alone [38]. This could be, hypothetically, due to the liberation of oxytocin that previous studies interpreted as a consequence of human-animal interaction [25,63,64]. Thus, the activation of the oxytocin system has been proposed to have a key role in the majority of these reported psychological and psychophysiological effects of AAT [25]. However, due to contradictory findings [26], further studies should confirm the results.

Another theoretical mechanism of AAT suggested by Kruger and Serpell [62] is social mediation, which states that the animal may mediate the interaction among patients and between the patient and the therapist. This, along with the known improvements in self-esteem and self-efficacy [18,19,20], may explain the expected effectiveness of the program in adolescents with eating disorders.

Regarding animal welfare, dogs participating in AAT may be even positively affected in interventions that are carefully designed when comparing their state before and after the session [65,66]. Thus, the well-being of dogs will be a priority in the current study. In this regard, different measures will be established, such as feeding the dog before training, limiting the time of training or ensuring rest between sessions. Additionally, the handler and the dog will live together to ensure that the handler adequately identifies and interprets the body language of the dogs during the sessions, reducing the possibility of suffering from stress, which may be experienced during AAT sessions [67,68].

### Limitations

This pilot study has several limitations that must be disclosed. First, the uncertain health conditions in Spain due to COVID-19 defines the maximum number of participants in each group and may change during the conduction of the study. Furthermore, the use of the mask is always mandatory (at least at the beginning of the study), which may affect the comfort and the communication between patients and between the patient and the staff. In addition, the use of face masks is an important limitation due to the influence of human facial expression in the relationship between participants and dogs [69]. In this regard, dogs are highly attentive to human facial expressions and can discriminate different emotions such as happiness [70] or anger [71]. This is important, as dogs can adjust their behavior according to the emotional signals of others [72]. Thus, when the COVID-19 restriction allows, the face mask will be removed.

Another limitation will be the lack of randomization and double-blinding, as participants will know whether they are in the intervention or control group. Additionally, this activity’s performance does not imply the cessation of other therapies, treatments, or activities that the patient is receiving. Therefore, it would be impossible to isolate the effect of these therapies in the adolescents completely. Lastly, the relatively small sample size and the large number of variables that we will assess could lead to insufficient statistical power to reach the significance level, so future studies must contrast the preliminary findings of this study. 

## 4. Conclusions

The current pilot-controlled trial will be the first to evaluate the effects of dog-assisted therapy on the physical and mental health of adolescents suffering from eating disorders. Significant improvements in the main symptoms of participants may be expected based on the known benefits of AAT on self-esteem, stress, and self-control in different populations. Although the intervention is focused on the improvement of adolescents’ health, animal welfare will also be adequately ensured.
